# Prediction of Brain Connectivity Map in Resting-State fMRI Data Using Shrinkage Estimator

**DOI:** 10.32598/bcn.9.10.140

**Published:** 2019-03-01

**Authors:** Atiye Nazari, Hamid Alavimajd, Nezhat Shakeri, Mohsen Bakhshandeh, Elham Faghihzadeh, Hengameh Marzbani

**Affiliations:** 1. Department of Biostatistics, Faculty of Allied Medical Sciences, Shahid Beheshti University of Medical Sciences, Tehran, Iran.; 2. Department of Radiology Technology, Faculty of Allied Medical Sciences, Shahid Beheshti University of Medical Sciences, Tehran, Iran.; 3. Department of Biomedical Engineering and Medical Physics, School of Medicine, Tehran University of Medical Sciences, Tehran, Iran.; 4. Neural Engineering Research Center, Noorafshar Hospital, Tehran, Iran.

**Keywords:** Resting-State fMRI, Functional connectivity, Shrinkage estimator, Mean Squared Error, Seed-based correlation analysis

## Abstract

**Introduction:**

In recent years, brain functional connectivity studies are extended using the advanced statistical methods. Functional connectivity is identified by synchronous activation in a spatially distinct region of the brain in resting-state functional Magnetic Resonance Imaging (MRI) data. For this purpose there are several methods such as seed-based correlation analysis based on temporal correlation between different Regions of Interests (ROIs) or between brain’s voxels of prior seed.

**Methods:**

In the current study, test-retest Resting State functional MRI (rs-fMRI) data of 21 healthy subjects were analyzed to predict second replication connectivity map using first replication data. A potential estimator is “raw estimator” that uses the first replication data from each subject to predict the second replication connectivity map of the same subject. The second estimator, “mean estimator” uses the average of all sample subjects' connectivity to estimate the correlation map. Shrinkage estimator is made by shrinking raw estimator towards the average connectivity map of all subjects' first replicate. Prediction performance of the second replication correlation map is evaluated by Mean Squared Error (MSE) criteria.

**Results:**

By the employment of seed-based correlation analysis and choosing precentral gyrus as the ROI over 21 subjects in the study, on average MSE for raw, mean and shrinkage estimator were 0.2169, 0.1118, and 0.1103, respectively. Also, percent reduction of MSE for shrinkage and mean estimator in comparison with raw estimator is 49.14 and 48.45, respectively.

**Conclusion:**

Shrinkage approach has the positive effect on the prediction of functional connectivity. When data has a large between session variability, prediction of connectivity map can be improved by shrinking towards population mean.

## Highlights

- Resting-state functional MRI scans with large session-to-session variability.
- Shrinkage estimators improve the prediction of each subject-specific functional connectivity maps.
- Using the shrinkage method has the advantage of analyzing big data with numerous variables and low observations.
- Shrinkage estimator can make it possible to cluster the brain more reliably because it provides a more reliable estimation of the functional connectivity map.

## Plain Language Summary

High variability of the acquired data and short-term scans in Functional magnetic resonance imaging studies are two significant challenges of data analysis, causing unreliable results. Shrinkage approach, in functional connectivity studies, presents reliable estimators considering the mentioned challenges. Comparing the results of classic estimators to the result of shrinkage estimators, it is found that shrinkage estimators provide more reliable and precise results. Moreover, because of creating a reliable functional connectivity map, any further analysis would be better in terms of reliability.

## 1. Introduction

Functional Magnetic Resonance Imaging (fMRI) is one of the valuable instruments to discover the function of the human brain. The fMRI is a non-invasive method that uses Blood Oxygen Level Dependent (BOLD) contrast mechanism (Daliri & Behroozi, 2012). In recent years, lots of researchers studied the patterns of brain functional connectivity (Behroozi, Daliri, & Boyaci, 2011; Ghaderi et al., 2017; Sadeghi et al., 2017). Functional connectivity focuses on how brain voxels and regions interact and function with each other.Some studiesare conducted on Resting-State (rs)-fMRI data to understand patterns ofbrainconnectivity and their role in brain diseases and disturbances (Zhang, Guindani, & Vannucci, 2015).In the resting-state imaging, without the stimulus, the subject is requested to lie down in the scanner device and not to move until the end of the imaging time (Daliri & Behroozi, 2013). Low-frequency fluctuations (<0.1 Hz) are observed in resting-state networks (Biswal et al., 1995).

Functional connectivity implies temporal correlation of the BOLD signals between voxels or Regions of Interest (ROIs) (Zhang et al., 2015). There are various analytical methods such as clustering (Cordes et al., 2002), partial correlation (Cribben et al., 2012; Varoquaux et al., 2010), independent component analysis and principal component analysis (Andersen, Gash, & Avison, 1999; Calhoun et al., 2001; McKeown et al., 1998). Some methods explore the dynamic functional connectivity, since healthy brain function may show rich dynamics over the course of time (Borumandnia et al., 2017).

Seed-based correlation analysis is one of the most widely used methods in functional connectivity analysis that is according to the temporal correlation between ROIs or between voxels in an ROI (Biswal et al., 1995; Fox et al., 2005). In new studies, the estimation of restingstate functional connectivity in aseed-based correlation analysis is improved by shrinkage approach (Shou et al., 2014). This estimator is used in scan-rescan rs-fMRI data to predict the functional connectivity by shrinking the subject-specific estimator towards the average connectivity maps of all subjects (Shou et al., 2014).

Due to large variability in subject-specific data, the results of short-term data usually tend to be highly unstable. One way to achieve a reliable estimate for each subject is to increase the time of brain imaging from a standard time 5–10 minutes to 30–60 minutes (Cohen et al., 2008). This gives the analyst more information, but this approach has limitations. A large number of rs-fMRI scans with a 5–10 minute imaging time were collected in the past and contained valuable information that is thus excluded. The impossibility of long-term scans of children, elderly, and sick people, and high cost of the brain imaging are leading obstacles (Mejia et al., 2015). Mejia et al. (2015). evaluated the effect of shrinkage estimators in simulated data with different lengths of time. In terms of reliability, shrinkage estimation of short-term data with 200 time and subject-specific estimation of a longterm data with 1000 time points have the same results

In the current study, shrinkage approach was applied to 21 healthy subjects (7–12 years old; 11 females and 10 males) with two scanning sessions to estimate correlation maps of the second replication using the first replication correlation coefficient. Each scanning session had 74-time points and the current study aimed at investigating the advantage of shrinkage approach to improve the prediction of functional connectivity in very short rs-fMRI scans.

## 2. Methods

Let Y_ij_(v, t) denote fMRI time series for each voxel of ROI at time t=1, …, T, for subject i=1, …, I, scanning session j=1, …, J. In the current study it was as follows: I=21, J=2, T=74. Seed time course is defined as:

(1)
$$Y_{\mathrm{ij}}^{s}(t)=\frac{1}{\left|s\right|}\sum_{v\in S}Y_{\mathrm{ij}}(\text{v},\;\text{t})$$,

where S and |S| are the collection of voxels and number of voxels in ROI, respectively. Seed-based correlation map is defined as the correlation between Y_ij_(v, t) and Y^S^_ij_(t):

(2)
$$W_{\mathrm{ij}}(\text{v})=\frac{\sum_{t=1}^{T}\left\{{\text{Y}}_{\mathrm{ij}}(\text{v},\text{t})-{\bar{\text{ Y }}}_{\mathrm{ij}}(\text{v},.)\right\}\left\{Y_{\mathrm{ij}}^{S}(t)(.)\right\}}{\sum_{t=1}^{T}{\left\{{\text{Y}}_{\mathrm{ij}}(\text{v},\text{t})-{\bar{\text{ Y }}}_{\mathrm{ij}}(v,.)\right\}}^{2}\sum_{t=1}^{T}{\left\{Y_{\mathrm{ij}}^{s}(t)-{\bar{Y}}_{\mathrm{ij}}^{s}(.)\right\}}^{2}}$$,

where Y̅_ij_ (v,.) is the average of Y_ij_ (v, t), and Y^S^_ij_(.) is the average of Y^S^_ij_ (t) over time (Shou et al., 2014). Equation (2) shows that the connectivity map is not dependent on time and can be calculated for each subject, each replication, and all voxels in ROI.

The goal was to predict second replication connectivity map, W_i2_ (v), of each subject using the first scanning session information. The connectivity map from the first replication of each subject can be considered as the estimation of second replication for the same subject, Ŵ^R^_i2_ (v)=W_i1_ (v), named “raw estimator”. The second estimator is “mean estimator” that uses the mean of the first replication connectivity map of all subjects in the study:

(3)
$${\hat{W}}_{i2}^{M}(v)=\frac{1}{I}\sum_{i=1}^{I}W_{i1}(\text{v}),i=1,...,21.$$

Therefore, the results are the same for all subjects. The third estimator is “shrinkage estimator” that shrinks raw estimator towards the average of the first replication connectivity map of all subjects (Shou et al., 2014).

For shrinking, Fisher Z transformation was employed as Equation (4) to normalize the correlation values, W_ij_ (V) (Shou et al., 2014).


(4)
$$\hat{V_{\mathrm{ij}}}=\frac{1}{2}\text{log}\frac{1+W_{\mathrm{ij}}(v)}{1-W_{\mathrm{ij}}(v)}$$

Then shrinkage estimator was performed according to Equation (5) on transformed coefficient (Shou et al., 2014).

(5)
$${\hat{V}}_{i2}^{SH}(v)=\lambda (v)*{\hat{V}}_{2}^{M}(v)+\left\{1-\lambda (v)\right\}*{\hat{V}}_{i2}^{R}(v)$$,

where:

(6)
$$\begin{array}{l} {\hat{V}}_{i2}^{R}(\text{v})=V_{i1}(\text{v})\\ {\hat{V}}_{2}^{M}(v)=\frac{1}{I}\sum_{i=1}^{I}V_{i1}(v) \end{array}$$

and *λ (v)* are shrinkage parameters that can take a value in [0, 1] (James & Stein, 1961; Shou et al., 2014).

Finally, to achieve the original scale of correlation, in-verse Fisher Z transformation was applied as Equation (7) to the shrinkage estimator (Shou et al., 2014).


(7)
$${\hat{W}}_{\mathrm{ij}}^{SH}(v)=\text{tanh}({\hat{\text{V}}}_{\mathrm{ij}}^{SH}(v))$$

If λ (v)=1, then raw estimator V̂^R^_i2_ (V) is completely unreliable and shrinkage estimator is reduced to the mean estimator. If λ (v)=0, the raw estimator is completely reliable and shrinkage estimator is equal to raw estimator, and no shrinkage occurs towards the average correlation map. Data are not usually reliable: hence, shrinking is a good option to improve prediction. Shou et al. (2013) reported λ (v)=λ=0.1 based on their data for shrinkage parameters of all voxels since this value is close to the average reliability of voxels. The optimized value of λ (v) can be calculated based on the replication data. The λ (v) is considered as reliability or Intra-class Correlation Coefficient (ICC) for connectivity map of each voxel (Shou et al., 2014).

To estimate λ (v), classical measurement error model for replication study is defined as:

(8)
$$V_{\mathrm{ij}}(v)=X_{i}(v)+{\text{U}}_{\mathrm{ij}}(v)$$,

where X_i_ (v) is true unobserved correlation coefficient for subject i, and U_ij_ (v) is the measurement error for subject i, replication j (Carroll et al., 2006).

It was assumed X_i_ (.) and U_ij_ (.) were uncorrelated with E (X_i_ (v))=μ_x_ (v) and E (U_ij_ (v))=0, therefore, the Best Linear Unbiased Estimator (BLUE) of X_i_ (v) was defined as Equation (9) (Shou et al., 2014):

(9)
$${\hat{\text{V}}}_{i2}^{SH}(v)=\hat{X_{i}}(v)=\lambda (v)*{\hat{V}}_{2}^{M}(v)+\left\{1-\lambda (v)\right\}*{\hat{V}}_{i2}^{R}(v)$$,

where:

(10)
$$\lambda (v)=\frac{\mathrm{Var}\left\{X_{i}(v)\right\}}{\mathrm{Var}\left\{X_{i}(v)\right\}+\mathrm{Var}\left\{{\text{U}}_{i1}(v)\right\}}$$

Variance of X_i_ (v) and U_ij_ (v) can be estimated based on the data as:

(11)
$$\begin{array}{l} D_{i}(v)=V_{i2}(v)-V_{i1}(v)\\ \mathrm{Var}\left\{U_{\mathrm{ij}}(v)\right\}=\frac{1}{2}Var_{i}\left\{D_{i}(v)\right\}=\frac{1}{2(\text{I}-1)}\sum_{i=1}^{I}{\left\{{\text{D}}_{i}(v)-\bar{\text{D}}(v)\right\}}^{2}\\ \mathrm{Var}\left\{{\text{V}}_{\mathrm{ij}}(v)\right\}=\frac{1}{J(\text{J}-1)}\sum_{j=1}^{J}\sum_{i=1}^{I}\left\{V_{\mathrm{ij}}(\text{v})-{\bar{V}}_{j}(\text{v})\right\}\\ \mathrm{Var}\left\{{\text{X}}_{i}(v)\right\}=\mathrm{Var}\left\{{\text{V}}_{\mathrm{ij}}(v)\right\}-\mathrm{Var}\left\{{\text{U}}_{\mathrm{ij}}(v)\right\} \end{array}$$,

where D̅ (v) and V̅_i_ (v) are the average of D_i_ (v) and V_ij_ (v), respectively. Estimating λ (v) and putting in Equation (4), shrinkage estimator is resulted (Mejia et al., 2015).

The performance of the three estimators is evaluated using Mean Square Error (MSE) criterion (Lehmann & Casella, 2006). The small value of MSE shows that the estimated value is close to true value. Therefore, the estimator with the lowest MSE among the others is the best one.


(12)
$$MSE=E{\left({\hat{\text{W}}}_{i2}(\text{v})-{\hat{\text{W}}}_{i2}(\text{v})\right)}^{2}$$

Ŵ_i2_ (v) is estimated as the connectivity map by each of the three methods, and W_i2_ (v) is the true value of obtained correlation in the second replication (Haman & Valenta, 2013).

The brain images used in the current study were related to a global competition called “ADHD-200 Preprocessed”^1^ in 2011, where its pre-processed source is free available. A variety of ways, including Athena pipeline^2^ are used to process these images. This method is the combination of processes performed by imaging software of FSL and AFNI (Bellec et al., 2017). Among this dataset, 21 healthy subjects between 7 to 12 years old and with 2 scanning sessions were randomly selected.

For pre-processing, structural images of skull bone tissue and background are removed from images, and White Matter (WM), Gray Matter (GM), and Cerebrospinal Fluid (CSF) regions are segmented. The binary mask is made up of white and gray matter regions. Skull-off images are mapped using a linear transformation to the Montreal Neurological Institute space (MNI). The resolution is changed to 1×1×1 mm, and the made masks are applied and the brightness of the images is normalized. Gaussian filter with 6 mm FWHM was used to smooth the images (Bellec et al., 2017).

Functional preprocessing steps were performed (e.g. removing the first four volumes, slice timing correction, motion correction, removing nuisance variance), data were also transformed to MNI space at 4×4×4 mm^3^ resolution, temporally filtered using 0.009-0.08 pass-band filter, and spatially smoothed (6-mm kernel) Bellec et al., 2017

The current study employed seed-based correlation analysis method to evaluate the estimation of functional connectivity of the second replication connectivity map. In this method, the precentral gyrus was selected as ROI. For the first time, Biswal et al. (1995) examined the analysis of brain function in this ROI. The mask of the pre-central regions were prepared using the WFU PickAtlas toolbox in MATLAB R2014a software (Maldjian, Laurienti, & Burdette, 2004; Maldjian et al., 2003). All brain images were mapped to type 2 Eve Atlas in the SPM12 (Oishi et al., 2009). Other calculations were performed in MATLAB R2014a software.

## 3. Results

**Figure 1.**
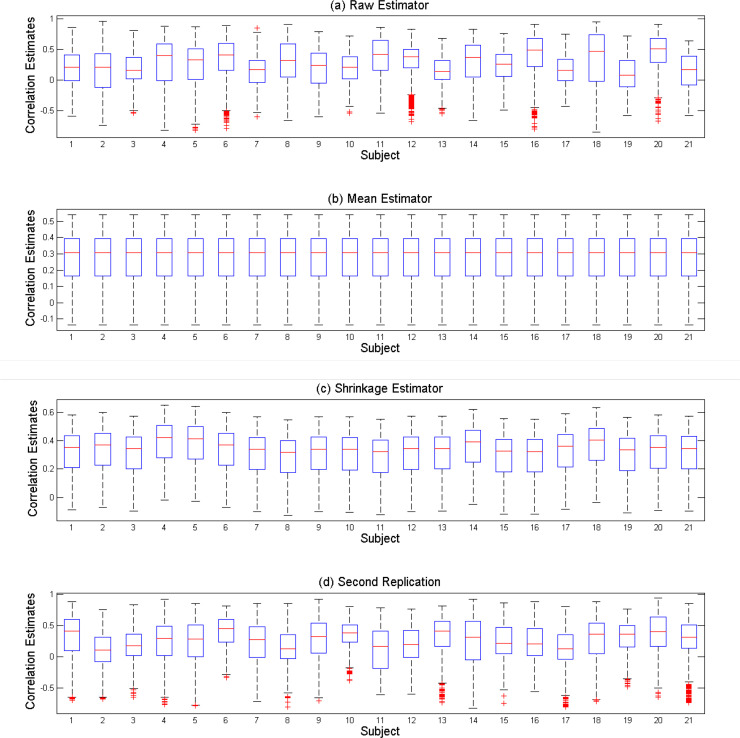
Distribution of correlation coefficients for each subject Boxplot of raw correlation estimates (a); mean correlation estimates (b); shrinkage correlation estimates (c); and the correlation coefficient of second replication data (d); Boxplots show the distribution of correlation coefficients of voxels of the precentral region for each subject. Red points are the outlier.

Seed-based correlation map of the second replication was estimated by raw, mean, and shrinkage methods for each voxel of the precentral region and for the 21 subjects. Figure 1 shows the distribution of correlation coefficients for each subject. Red points are the outliers. Mean estimation of connectivity map was the same for all subjects; hence, the corresponding boxplot for all subjects had the same pattern (Figure 1 b). As an example, the results of the subject 18 were considered. For this subject, the raw estimator predicted that the correlation coefficients were more scattered (Figure 1 b) than the values obtained from the second replicate of this subject (Figure 1 d).

**Figure 2.**
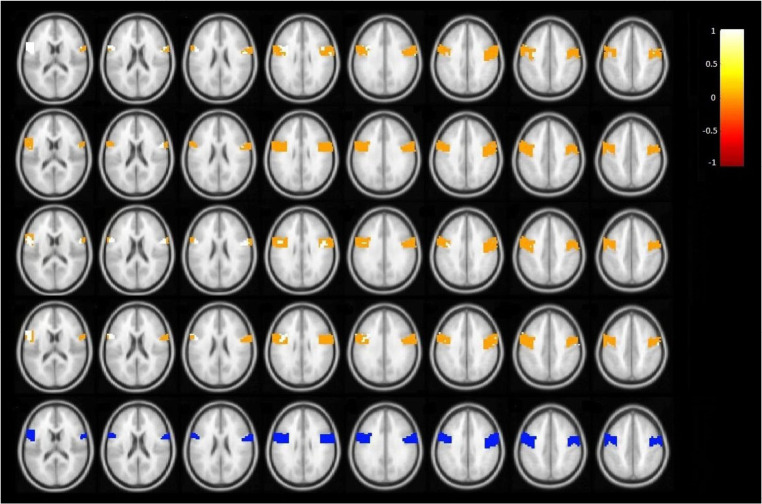
Connectivity maps of the specific subject with similar connectivity map to the average of all subjects using different methods First row: Raw estimator, Second row: Mean estimator, Third row: Shrinkage estimator, Fourth row: True correlation coefficient of the second replication, Fifth row: The mask of the precentral gyrus; The white and yellow colors indicate the positive correlation and the orange and red colors represent a negative correlation. Each column shows the results in a different axial slice.

The shrinkage estimator showed a similar pattern along the box and the range of correlation coefficients (Figure 1 c). All three methods can predict negative skew inthe distribution of truecorrelation coefficients. Figure 2 displays the correlation map in eight different axial slices for a particular subject. Connectivity map of this subject was almost similar to the average of the connectivity map of all subjects. The white and yellow colors indicate the positive correlation and the orange and red colors represent a negative correlation. The last row is the mask of the precentral region and the voxels of this region are displayed in blue (Figure 2 e). The colors showed that the shrinkage estimator (Figure 2 c) had closer correlation patterns to the map of the connection derived from the second repetition (Figure 2 d).

**Figure 3.**
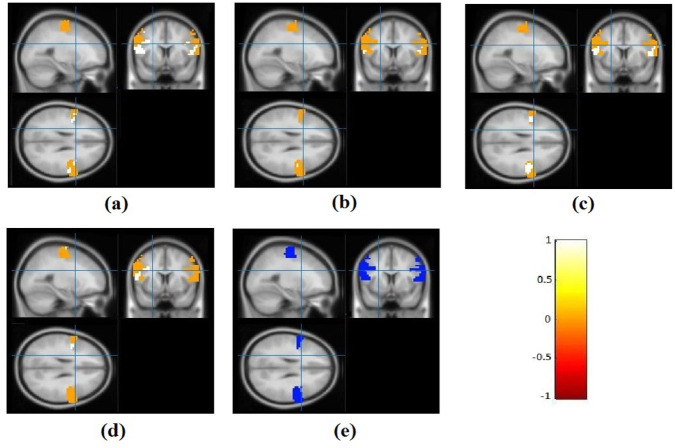
Connectivity maps in sagittal, coronal, and axial views of the specific subject with similar connectivity map to the average of all subjects using different methods (a) Raw estimator; (b) Mean estimator; (c) Shrinkage estimator; (d) Correlation coefficient of second replication; and (e) The mask of the precentral gyrus; The white and yellow colors indicate the positive correlation and the orange and red colors represent a negative correlation.

Figure 3 also shows the results of the specific subject in certain spatial coordinates in the three views. In other views, the shrinkage estimator (Figure 3 c) shows the nearest correlation map to the true values (Figure 3 d). Using the Mean Squares Error (MSE) criterion, the performance of the three estimators to predict the second replication connectivity map was evaluated. Table 1 reports MSE from these three methods to each subject. For all subjects, the MSE of mean estimator was lower than the raw estimator and the MSE of shrinkage estimator was the lowest. The minimum and maximum MSE of raw estimator were 0.1035 and 0.4163, respectively. By the shrinkage method, MSE decreased to a minimum of 0.0629 and a maximum of 0.1629. The average MSE of raw, mean, and shrinkage estimators was 0.2169, 0.1118, and 0.1103, respectively. Also, the decrease of MSE for mean and shrinkage estimator in comparison with MSE of the raw estimator was positive for all people indicating a better prediction of the true correlation coefficients of the second repetition using the mean and shrinkage estimators. On average, the reduction in MSE was 48.45% for the mean estimator and 49.14% for shrinkage estimator.

**Table 1.** Average of MSEs over all voxels for raw, mean, and shrinkage estimators for each subject and the reduction in MSE for mean and shrinkage estimators in comparison with the raw estimator

**Subject**	**Raw**	**Mean**		**Shrinkage**	

	**MSE**	**MSE**	**Red. %**	**MSE**	**Red. %**
1	0.2183	0.1342	38.50	0.1336	38.36
2	0.1929	0.0981	49.15	0.0975	49.43
3	0.1313	0.0854	34.95	0.0851	35.13
4	0.2520	0.1127	55.25	0.1114	55.78
5	0.2313	0.1137	50.80	0.1125	51.03
6	0.2317	0.1639	29.23	0.1629	29.66
7	0.1440	0.0985	33.45	0.0955	33.68
8	0.2052	0.1082	47.24	0.1081	47.30
9	0.2149	0.1241	42.22	0.1237	42.43
10	0.1725	0.1033	40.06	0.1030	40.26
11	0.4163	0.1572	62.22	0.1570	62.28
12	0.1961	0.0876	55.34	0.0873	55.49
13	0.2199	0.1411	35.83	0.1406	36.03
14	0.2625	0.1566	38.02	0.1553	38.52
15	0.1035	0.0641	38.07	0.0640	38.11
16	0.2858	0.0630	77.93	0.0629	77.99
17	0.1317	0.1157	12.11	0.1151	12.60
18	0.3384	0.1032	69.48	0.1022	69.79
19	0.1861	0.0753	59.49	0.0751	59.62
20	0.2052	0.1372	33.13	0.1366	33.42
21	0.2025	0.1074	46.93	0.1070	47.16
Average	0.2169	0.1118	48.45	0.1103	49.14

**Figure 4.**
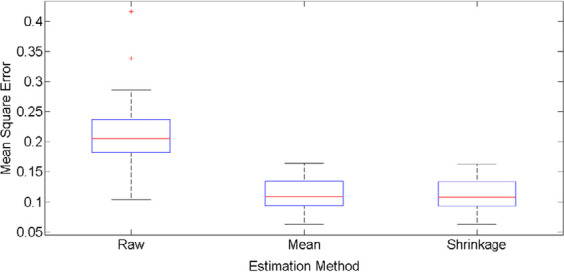
The boxplots of MSE for 3 different prediction methods; raw, mean, and shrinkage correlation estimates

Figure 4 shows the MSE boxplot of all three methods. The raw estimator had larger MSE values and, with regard to box length, a more dispersed distribution. The MSE values for the mean and shrinkage estimators were almost proportional to the amount and dispersion and less than those of the raw estimator. The median MSE of raw, mean, and shrinkage estimators was 0.2053, 0.1083, and 0.1082, respectively. Due to the fact that the median line was not in the middle of the box, the distribution of MSE values for the three estimators was skew to the right. There were two outliers of 0.4163 and 0.3384 in the distribution of MSE of the raw estimator, which were respectively related to the eleventh and eighteenth subjects.

## 4. Discussion

The current study employed a shrinkage approach to improve the estimation of functional connectivity in the seed-based correlation analysis. The test-retest rs-fMRI data with 74 time points in 21 healthy subjects were applied. The precentral gyrus was selected as an ROI. On Fisher Z transformation correlation coefficient, the average MSE for raw, mean, and shrinkage estimators was 0.2169, 0.1118, and 0.1103, respectively. The prediction performance improved 49.14% and 48.45% by shrinkage and mean estimators in comparison with that of the raw estimator. The prediction of the functional connectivity of all subjects improved.

Shou et al. (2014) also performed this procedure in the 20-subject rs-fMRI test-retest data with 210 time points. Applying shrinkage and mean estimators on fisher transformed coefficients reduced MSE by 30% and 25%, respectively. The shrinkage estimator improved the prediction of 18 out of 20 subjects.

Mejia et al. (2015) used the shrinkage approach to estimate the similarity matrix. The implementation of the shrinkage estimator on the Fisher-transformed and original correlation coefficients improved the reliability of the results 29% and 26%, respectively.

The number of people in the study does not change the results of the raw estimator, since this estimation does not depend on the information of other sample subjects, but the performance of shrinkage estimator is influenced by the number of people in the study. By simulating the different sample sizes, the shrinkage estimator provides the best result with 20 subjects and no significant improvement in prediction is achieved with increasing the subjects (Mejia et al., 2015).

A shrinkage approach is used to improve the results of many classical estimators. In the current study investigated the advantage of analyzing big data with many variables and low observations. When rs-fMRI scans are very short with large session-to-session variability, the estimation of each subject-specific connectivity maps can be improved by shrinking towards the average of other subjects. Also, the correlation matrix of voxels used as a similarity matrix of clustering methods can be estimated by shrinkage method. Due to the fact that the shrinkage estimator provides reliable estimates of the correlation between the time series of the voxels, its application makes it possible to cluster the brain more reliably.

## Ethical Considerations

### Compliance with ethical guidelines

There is no ethical guidelines; Because the data has been downloaded.

## Footnotes

http://www.nitrc.org/frs/?group_id=383

http://www.nitrc.org/plugins/mwiki/index.php/neurobureau: AthenaPipeline
